# Sevoflurane Postconditioning Attenuates Hepatic Ischemia-Reperfusion Injury by Limiting HMGB1/TLR4/NF-κB Pathway *via* Modulating microRNA-142 *in vivo and in vitro*

**DOI:** 10.3389/fphar.2021.646307

**Published:** 2021-04-16

**Authors:** Liying Xu, Feng Ge, Yan Hu, Ying Yu, Kefang Guo, Changhong Miao

**Affiliations:** Department of Anesthesiology, Zhongshan Hospital Fudan University, Shanghai, China

**Keywords:** hepatic ischemia-reperfusion injury, sevoflurane, postconditioning, microRNA-142, HMGB1/TLR4/NF-κB pathway

## Abstract

Preconditioning of sevoflurane (Sevo) has been demonstrated to protect the liver from ischemia/reperfusion (I/R) injury. However, it is unknown whether it has hepatoprotective when given at the onset of reperfusion (postconditioning), a protocol with more clinical impact. The present study aimed to explore the hepatoprotective effects of Sevo postconditioning against hepatic IR injury *in vivo* and *in vitro* and the possible mechanisms. Using a mouse model of hepatic I/R, Sevo postconditioning significantly improved hepatic injury after reperfusion, as demonstrated by reduced AST, ALT, and LDH serum levels and reduced histologic damage in liver tissues. Furthermore, Sevo postconditioning could suppress the apoptosis, inhibit oxidative stress and inflammatory response in liver tissue of HIRI mice, as well as improve the survival rate of HIRI mice. Through analyzing GSE72314 from the gene expression omnibus (GEO) database, it was demonstrated that microRNA (miR)-142 is downregulated by HIRI, which was reversed by Sevo treatment. Further investigation showed that agomiR-142 injection could enhance the hepatoprotective effects of Sevo postconditioning on I/R injury, while antagomiR-142 reversed these effects in mice. Notably, high mobility group box 1 (HMGB1), an important inflammatory factor, was directly targeted by miR-142 in hepatic cells, and we further found that Sevo could inhibit the expression of HMGB1 through up-regulating miR-142 expression in HIRI mice model. In addition, we found that I/R injury induced the activation of TLR4/NF-κB inflammatory pathway was partially suppressed by Sevo postconditioning, and miR-142 mediated the regulatory role of Sevo postconditioning. In line with the *in vivo* results, Sevo treatment improved the cell viability, inhibited cell apoptosis, oxidative stress and inflammatory response *in vitro* HIRI model, while these effects were reversed by antagomiR-142 transfection. Collectively, our findings demonstrated that Sevo postconditioning counteracts the downregulation of miR-142 provoked by I/R, in turn decreased the expression of HMGB1, blocking TLR4/NF-κB pathway activation, thus improving hepatic I/R injury. Our data suggest that Sevo may be a valuable alternative anaesthetic agent in liver transplantation and major liver surgeries.

## Introduction

Hepatic ischemia/reperfusion injury (HIRI) is a pathophysiological condition post major liver surgeries or transplantation, which results in severe liver damage. The main mechanisms have been implicated in the hepatic I/R injury process, including oxidative stress, inflammatory responses, and hepatocyte apoptosis (Vardanian et al., 2008; Haga et al., 2009). Ischemic preconditioning (sevoflurane are administered upon the occurrence of ischemia) is one of the methods used to attenuate IR injury (Theodoraki et al., 2016). The main limitation of preconditioning in the clinical context is that they must be initiated before the ischemic insult, which is not always predictable. In recent years, ischemic postconditioning was considered as a novel approach to minimize IR injury, which is defined as repeated brief cycles of ischemia and reperfusion after the prolonged period of ischemia and before the sustained reperfusion (de Rougemont et al., 2009; Santos et al., 2010). This technique is operative and easy to perform in patients undergoing liver surgeries or transplantation.

Sevoflurane (Sevo), an inhaled anesthetic, has protective effects on organ I/R injury in the lung and liver (Rancan et al., 2014; Cavalcante et al., 2015; Ohsumi et al., 2017). Sevo preconditioning and postconditioning have equally protective effects against hepatic I/R injury, but the latter decreases liver injury with better patient outcome than Sevo preconditioning in clinical therapy (Wang et al., 2010; Zhang et al., 2011; Beck-Schimmer et al., 2012; Shiraishi et al., 2019). Sevo postconditioning protects the liver from I/R injury by multiple mechanisms that involve apoptosis, inflammatory response and reactive oxygen species (ROS) (Beck-Schimmer et al., 2018; Figueira et al., 2019). However, the underlying biological pathophysiology need to be further explored, which encouraged us to investigate the molecular mechanisms and pathways.

MicroRNAs (miRNAs) are a group of endogenous, small noncoding RNAs with 19∼22 nucleotides in length, which have been certified as essential regulators of gene expression at posttranscriptional levels (He and Hannon, 2004). Abnormally expressed miRNAs have been identified in pathological events associated with hepatic I/R injury. For example, Shen et al. found that miR-24-3p protected against hepatic I/R injury in mice through the repression of STING signaling pathway (Shen et al., 2020). Mou et al. reported that miR-128-3p inhibition could alleviate liver I/R injury through suppressing Rnd3/NF-κB axis in mice (Mou et al., 2020). Zhang et al. demonstrated that miR-449b-5p attenuated hepatic I/R injury by targeting HMGB1 (Zhang et al., 2019a). More recently, the protective effects of Sevo preconditioning against I/R injury have been associated with the regulation of many miRNAs (Zhao Y.-B. et al., 2019; Liao et al., 2019). Wu et al. showed that Sevo preconditioning ameliorated hepatic I/R injury in mice through miR-200c downregulation (Wu et al., 2016), but few studies focus on the miRNA regulation mechanism of Sevo postconditioning on hepatic I/R injury.

In the present study, the aim was to assess the beneficial effects of Sevo postconditioning against hepatic I/R injury in mice model. Furthermore, the molecular mechanisms involved in Sevo postconditioning in hepatic I/R injury were investigated, giving emphasis to miRNA regulation. Collectively, our findings will provide new insight into the protective effect of Sevo postconditioning on hepatic injury.

## Methods and Materials

### Experimental Animals

Male BALB/c mice (18–22 g) were obtained from the Shanghai SLAC Laboratory Animal Co. Ltd. (Shanghai, China). All mice were housed under standard conditions (12 h light-dark cycle, 21 ± 2°C, ∼55% humidity) with free access to food and water. Animal procedures were approved by the Animal Care and Use Committee the Zhongshan Hospital Fudan University.

### Hepatic Ischemia-Reperfusion Injury

All mice were fasted overnight before ischemia-reperfusion operation. Mice were anesthetized with pentobarbital sodium (50 mg/kg) and then the abdomen was opened through a midline incision. All vessels were occluded for 30 min by clamping the portal vein, hepatic artery, and bile duct of the left and median, and then released for 2 h reperfusion. The harvested blood samples were centrifuged at 1500 g for 10 min to obtain serum, and then stored at −80°C until further examination. Liver tissues were snap frozen in liquid nitrogen or fixed in 10% formaldehyde.

### Experimental Design

All mice were randomly divided into 5 groups (*n* = 6 per group): the Sham group; HIRI group; Sevo + HIRI group; Sevo + HIRI + agomiR-142 group and Sevo + HIRI + antagomiR-142 group. The mice in the Sham group only underwent operation, without ischemia/reperfusion (I/R) treatments. The mice in the HIRI group were subjected to I/R. The mice in the Sevo + HIRI group were subjected to 30 min of ischemia, then post-treatment with inhalation of 2% Sevo for 2 h immediately at the onset of reperfusion. The mice in the Sevo + HIRI + agomiR-142/antagomiR-142 group were injected *via* tail vein with agomir-142 or antagomiR-142 (30 mg/kg) 24 h prior to ischemia, then post-treatment with inhalation of 2% Sevo for 2 h immediately at the onset of reperfusion. Agomir-142 or antagomiR-142 was injected into the mice using a glass micropipette (tipdiameter 20–40 µm) *via* tail vein injection. The dose of the agomiR-142/antagomiR-142 (30 mg/kg) was based on the results obtained from in sepsis model (Zhen and Chen, 2018). The agomiR-142, antagomiR-142 and negative controls were synthesized by GenePharma (Shanghai, China).

### Sevoflurane Treatment

According to the experimental protocol as previously described (Zhao Y.-B. et al., 2019), mice were placed in a 25 cm × 18 cm × 18 cm square box and exposed to either 2% Sevo (purity >99%, J&K chemical, Beijing, China) in a 30% oxygen carrier gas (balanced with nitrogen) or a carrier gas without Sevo for 2 h. The induction flow rates were 6 l/min for the first 5 min for induction. The concentrations of Sevo, oxygen and carbon dioxide in the chamber were detected at the chamber exit port by a gas monitor (PM 8060, Dräger, Gernany).

### Liver Function Analysis

The levels of AST and ALT were determined in blood samples using a VetTest 8008 automatic biochemical analyzer. The activity of LDH in serum samples was assayed using commercial kits (Jiancheng Biotechnology Co., Ltd., Nanjing, China).

### Histological Analysis

At the end of the procedures, liver tissue obtained from each animal was taken for histology examination. The samples were fixed in 10% formaldehyde buffered with phosphate, embedded in paraffin and then cut in 4 μm sections using a Leica SM 2010R microtome (Leica, Shanghai, China), followed by staining with hematoxylin and eosin (HE), including staining with 0.5% hematoxylin for 5 min followed by 0.5% eosin for 1 min at 25°C. The Suzuki score (criteria, 0–4) assesses liver damage and was used for grading of hepatic I/R injury based of the fixed tissues (Suzuki et al., 1993). All sections were examined with a light microscopy (Olympus BX microscope, Shinzyuku, Tokyo, 200× magnification).

### The Survival Experiments

The survival experiments were performed in all 4 groups of mice (the HIRI, HIRI + SEVO, HIRI + SEVO + agomir-142 and HIRI + SEVO + antagomir-142 groups; *n* = 10/each group). Mice were treated as aforementioned. Mice were monitored regularly, and survival was recorded over a period of 7 days.

### TUNEL Assay and Immunohistochemistry (IHC) Staining

Liver paraffin sections prepared as above described, were used to detect cell apoptosis by the DeadEnd^TM^ Colorimetric TUNEL System (Promega, United States) according to the manufacturer’s protocol. The number of TUNEL-positive cells in 30 sections per slide was counted to estimate the level of liver tissue apoptosis at 200× magnification as described previously (Hu et al., 2013).

Liver paraffin sections were also used for immunohistochemistry staining as routinely processed (Zhou et al., 2016). The sections were blocked with 10% bovine serum for 30 min, and then incubated overnight at 4°C with cleaved caspase-3 (cat. no. 9664, Cell Signaling Technology, Inc., 1:100). Subsequently, the sections were incubated with goat Anti-Rabbit IgG H&L (Alexa Fluor^®^488) (cat. no. ab150081; 1:2,000; Abcam). Finally, the immune-reactivity was visualized by staining with diaminobenzidine (DAB; D-5637, Sigma-Aldrich, Saint Louis, MO, United States) for 3 min and sections were counterstained with hematoxylin. All images were captured by using a light microscope (Olympus BX microscope, Shinzyuku, Tokyo, 200× magnification). Positively stained cells were observed and counted by the Image-Pro Plus image analysis management system (Media Cybernetics, Rockville, MD). Each slice was randomly selected five horizons to calculate positive cells.

### Enzyme-Linked Immunosorbent Assay (ELISA)

Serum cytokines IL-1β (cat no. 96-403), IL-6 (cat no. 96-407), IFN-α (cat no. 96-416), and IL-10 (cat no. 96-408) were detected by ELISA kits according to the kit instructions. All ELISA kits were obtained from Merck-Millpore, Billerica, MA, United States.

### Measurement of SOD and MDA Levels

The liver tissues were placed in cold saline (1: 10, w/v), homogenized with a homogenizer machine, and then centrifuged at 3000 r/min for 15 min to produce the supernatant for detecting the levels of superoxide dismutase (SOD) (cat no. S0103, Jiancheng Biotechnology Co., Ltd. Nanjing, China) and malondialdehyde (MDA) (cat no. S0131, Jiancheng Biotechnology Co., Ltd. Nanjing, China) levels according to the respective instructions.

### miRNA Microarray Assay

Microarray dataset was obtained from GEO database (https://www.ncbi.nlm.nih.gov/geo/query/acc.cgi?acc=GSE72314) and the GEO accession number is GSE72314. GSE72314 dataset was based on Illumina HiSeq 2000 (*Mus musculus*) platform. GEO2R (www.ncbi.nlm.nih.gov/geo/geo2r/), an interactive web tool was applied to compare the samples in two different groups under the same experimental condition. Differentially expressed miRNAs (DE-miRNAs) were then identified based on the fold change. The heat map of DE-miRNAs was created using a method of hierarchical clustering by GeneSpring GX, version 7.3 (Agilent Technologies, California, United States).

### RNA Isolation and Quantitative RT-PCR

Total RNA was extracted from liver tissues with a miRNeasy Mini kit (Qiagen GmbH, Hilden, Germany). For detection of miRNA expression, cDNA was obtained from 10 ng RNA using Taqman MicroRNA Assays (Applied Biosystems) at 42°C for 1 h. For detection of mRNA expression, cDNA was prepared from 300 ng RNA using PrimeScript RT Master Mix (Takara) at 42°C for 1 h. The U6 gene was used as a reference control for miR-142-5p and GAPDH was used as a reference control for HMGB1. The RT-PCR reactions for the miRNAs and mRNA were analyzed using SYBR^®^ Premix Ex Taq™ (Takara Bio, Inc.) on an ABI Prism 7900 HT (Applied Biosystems). The primers used for were as follows: miR-125b, F 5′-GCC​CTC​CCT​GAG​ACC​TCA​A-3'; R: 5′-GTG​CAG​GGT​CCG​AGA​T-3′ miR-24, F, 5′-GCA​GAT​GGC​TCA​GTT​CAG​CAG-3'; R, 5′-GTG​CAG​GGT​CCG​AGG​T-3'; miR-142, F, 5′-GTC​GTA​TCC​AGT​GCA​GGG-3'; R, 5′-CGA​CGT​GTA​GTG​TTT​CCT​A-3'; miR-17, F, 5′- TCC​GTG​AGA​ACT​CAA​TTC​C -3′, R, 5'- \GAG​CAG​GGT​CCG​AGG​T-3'; miR-191; F, 5′-CGG​AAT​CCC​AAA​AGC​AGC​TG-3′, R: 5′-TGT​CGT​GGA​GTC​GGC​AAT​TG-3'; HMGB1, F, 5′-CTC​AGA​GAG​GTG​GAA​GAC​CAT​GT -3′, R, 5′-GGG​ATG​TAG​GTT​TTC​ATT​TCT​CTT​TC-3'; U6 F, 5′-GCT​TCG​GCA​GCA​CAT​ATA​CTA​AAA​T-3′, R, 5′-CGC​TTC​AGA​ATT​TGC​GTG​TCA​T-3ʹ; GAPDH F, 5ʹ-CAG​CCT​CAA​GAT​CAT​CAG​CA-3ʹ and R, 5ʹ-GTC​TTC​TGG​GTG​GCA​GTG​AT-3ʹ. Relative expression levels were calculated using the 2^−∆∆Ct^ method (Livak and Schmittgen, 2001).

### Cell Culture and Transfection

NCTC 1469 cell line (ATCC, Manassas, VA, United States) was maintained in DMEM/F12 containing 10% FBS (Gibco; Thermo Fisher Scientific, Inc.), and 1% penicillin and streptomycin (Sigma-Aldrich, St. Louis, MO, United States) at 37°C in a humidified 5% CO_2_ incubator. When NCTC 1469 cells grown to about 80% confluence in six-well plate, the cells were transfected with 100 nM agomiR-142, agomiR-NC, antagomiR-142 or antagomiR-NC at 37°C for 48 h, using Lipofectamine® 2000 (Invitrogen).

A cellular model of simulated I/R in BNL CL.2 cell line was used as previously described (Liu D. et al., 2018). BNL CL.2 cells were divided into the groups as follows: Control, H/R, H/R + Sevo, H/R + Sevo + antagomir-NC and H/R + Sevo + antagomir-142. Normal BNL CL.2 cells were used as the Control group. For the H/R group, BNL CL.2 cells (1 × 10^5^ cells/well) were cultured in an incubator with 95% N_2_ and 5% CO_2_ for 6 h; after the culture medium was subsequently replaced with a fresh 10%-FBS medium, the cells were cultured with 5% CO_2_ for another 6 h. The same process of ischemia-reperfusion was performed in cells of the H/R + Sevo group, except that they were pre-cultured in 5% CO_2_ with 2% Sevo for 1 h before culture in an incubator. For H/R + Sevo + antagomir-NC/antagomir-142 group, cells were transfected with antagomir-NC/antagomir-142 (100 nM) for 24 h, and then the same process was performed in H/R + Sevo group.

### Cell Viability

Cell viability was determined using cell counting Kit-8 (CCK-8), according to the manufacturer’s instructions (Dojindo, Kumamoto, Japan). Briefly, 10 µL of CCK-8 solution was added into each well (2 × 10^5^/well) and the cells were incubated for another 2 h at 37°C. The absorbance was detected at 450 nm using a microplate reader (Model 680; Bio-Rad Laboratories, Inc.).

### Caspase-3 Activity Assay

BNL CL.2 cells were seeded in 6-well plates at a density of 1 × 10^5^ cells/well for 24 h. After cells treatment as above mentioned, caspase-3 activity in cell lysates was determined using a Caspase-3 Activity Assay kit (Beyotime Institute of Biotechnology, China), according to the manufacturer's protocol.

### Measurement of Intracellular ROS

The BNL CL.2 cells were plated in 6-well culture plates at a density of 5 × 10^4^ cells/ml and then subjected to I/R for 24 h. After removing the medium, 1.5 ml of DCFH-DA (10.0 μM) was added at 37°C for 25 min, and then the samples were analyzed by fluorescence microscopy (Olympus, Tokyo, Japan) at 200× magnification.

### NF-κB Activity

The BNL CL.2 cells were plated in 6-well plates at a concentration of 5 × 10^4^ cells/well. The cells were allowed to attach overnight and then co-transfected with 20 ng of the pGL4.32 [luc2P/NF-κB-RE/Hygro] vector and 5 ng of the pRL-TK vector in each well (Promega Corporation). After 6 h, the cells were washed with PBS and then transfected with antagomir-142 for 24 h, followed subjected to subjected to I/R for 24 h. However, it should be noted that, before ischemic treatment, cells of each group were placed in a semi-airtight container and 2% Sevo was used for 2 h of pre-treatment. The cells were washed in PBS and harvested in 500 µL 1X passive lysis buffer. Luciferase activity was quantified using the Promega luciferase assay kit on a luminometer.

### Luciferase Reporter Assay

The predicted and mutated sequences targeting the 3′UTR of HMGB1 were amplified and cloned into the pGL3 vector (Promega Corporation, Madison, WI, United States). pGL3-HMGB1-3′UTR wild-type (Wt) and pGL3-HMGB1-3′UTR mutated (Mut) were synthesized by GenePharma*.* NCTC 1469 cells (1–2 × 10^5^ cells per well) were co-transfected with 0.2 µg pGL3-HGMB1-3′UTR Wt, 0.2 µg pGL3-HGMB1-3′UTR Mut and 100 nM agromiR-142 using Lipofectamine® 2000 (Invitrogen; Thermo Fisher Scientific, Inc.) for 24 h at 37°C. Luciferase activity was detected using the Dual-Luciferase Reporter Assay system (Promega Corporation). The pRL-TK vector was used as an internal control.

### Western Blot Analysis

Total protein was extracted from harvested liver tissues or cells using lysis buffer (Beyotime, Shanghai, China). The extraction and isolation of nuclear and cytoplasmic protein were performed according to the Nuclear and Cytoplasmic Protein Extraction Kit (cat no. P0027, Beyotime, Shanghai, China). The concentration of the protein was measured by BCA protein assay kit (Beyotime, Shanghai, China). 40 μg extracted protein samples from liver tissues and cells were transferred onto PVDF (Millipore) membranes. These membranes were blocked with 5% skim milk for 2 h at room temperature, and then incubated with primary antibodies against GPx (cat. no. 3286), p22phox (cat no. 27297), caspase 3 (cat. no. 9662), cleaved-caspase-3 (cat. no. 9664), TLR4 (cat. no. 14358), MyD88 (cat. no. 4283), nuclear-p-p65 (cat. no. 3033), p65 (cat. no. 8242), p-IκBα (Ser32 cat. no. 2859), IκBα (cat. no. 4812), IL-6 (cat. no. 12912), IL-10 (cat. no. 12163), IL-1β (cat. no. 12703), TNF-α (cat. no. 11948), HMGB1 (cat no. 6893), Histone 3 (cat. no 4499) and β-actin (cat. no. 4970) (All antibodies were obtained from Cell Signaling Technology, Inc. and the dilution was 1:1000) at 4°C overnight. After that, the blots were washed with TBST and further incubated at room temperature with secondary antibodies (cat. no. 7074; Cell Signaling Technology, Inc. 1:2,000) for 1 h. The protein bands were visualized using ECL kit (GE Healthcare) and quantified with ImageJ version 1.46 (Rawak Software, Inc. Munich, Germany).

### Statistical Analysis

Statistical analysis was performed using SPSS (version 13.0; SPSS, Inc.). Data were presented as means ± SD. One-way ANOVA followed by least significant difference post-hoc tests was used to analyze differences among multiple groups. A *p* value < 0.05 was considered significant. Survival studies were analyzed using the log rank test and the results are presented as the Kaplan–Meier curves. Each experiment was repeated at least three times.

## Results

### Sevo Postconditioning Attenuated Hepatic Injury Induced by I/R *in vivo*

**FIGURE 1:**
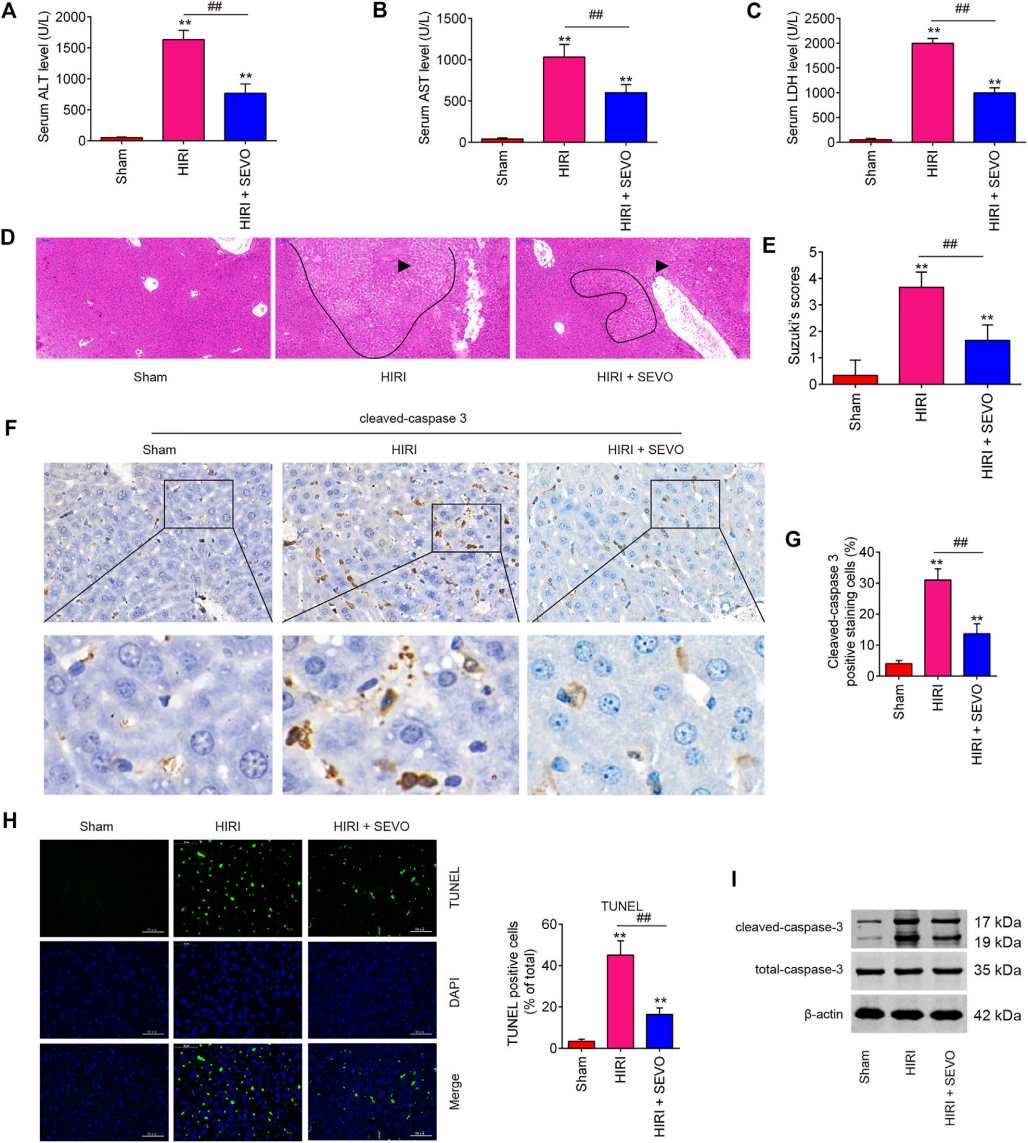
Sevoflurane postconditioning decreases hepatic I/R injury. Mice (*n* = 6/group) were subjected to ischemia, then post-treatment with inhalation of 2% Sevo for 2 h immediately at the onset of reperfusion. **(A)** Liver enzyme assays were performed in the three groups: sham, HIRI and HIRI + Sevo groups. **(A)**: Serum ALT levels; **(B)**: Serum AST levels; **(C)**: Serum LDH levels). **(D, E)** Liver damage induced by I/R was visualized using HE staining and was graded using the Suzuki score. The inflammatory cell infiltration is indicated with black arrow and necrotic areas is indicated with curve. **(F, G)** The expression of cleaved-caspase-3 was measured by IHC. **(H)** The apoptosis was determined by TUNEL staining assay. **(I)** The expression of cleaved caspase-3 was detected by Western Blot. All data were measurement data which were expressed as mean ± standard deviation and analyzed by one-way analysis of variance. **p < 0.01 vs. Sham group; ^##^ *p* < 0.01 vs. HIRI group.

Firstly, to evaluate the therapeutic effects of Sevo on hepatic I/R injury, mice were subjected to ischemia, then post-treatment with inhalation of 2% Sevo for 2 h immediately at the onset of reperfusion. High serum levels of AST, ALT and LDH, indicators of severe liver damage, were observed in the HIRI group compared with the Sham group, while the levels of these indicators were significantly reduced in the Sevo + HIRI group compared with HIRI group (Figures 1A–C). Liver damage induced by I/R was also visualized using HE staining and was graded using the Suzuki score, and the results indicated severe liver damage such as necrosis and inflammatory cell infiltration in HIRI group; however, Sevo postconditioning significantly attenuated liver damage compared with HIRI group (Figures 1D,E), which suggest that Sevo postconditioning could partially protect against hepatic I/R injury. Since the I/R-induced hepatic injury is partly due to induction of hepatocyte apoptosis (Quesnelle et al., 2015), the expression level of cleaved caspase 3 was measured by IHC and the results showed that the levels of cleaved caspase 3 expression in HIRI group were significantly increased compared with Sham group, while Sevo postconditioning attenuated the levels of caspase 3 induced by I/R (Figures 1F,G). Meanwhile, the hepatocyte apoptosis was determined by TUNEL assay. As shown in Figure 1H, the numbers of TUNEL-positive hepatocytes in HIRI group were higher than that in Sham group, whereas the numbers was significantly reduced in Sevo + HIRI group compared with HIRI group. In addition, similar results were also observed in the expression of cleaved-caspase-3 in liver tissues, as determined by Western Blot (Figure 1I). Collectively, these data suggests that Sevo postconditioning could alleviate I/R-induced hepatic injury in mice by inhibiting hepatocyte apoptosis.

### Sevo Postconditioning Exhibits Anti-oxidative and Anti-inflammatory Activities *in vivo*

**FIGURE 2:**
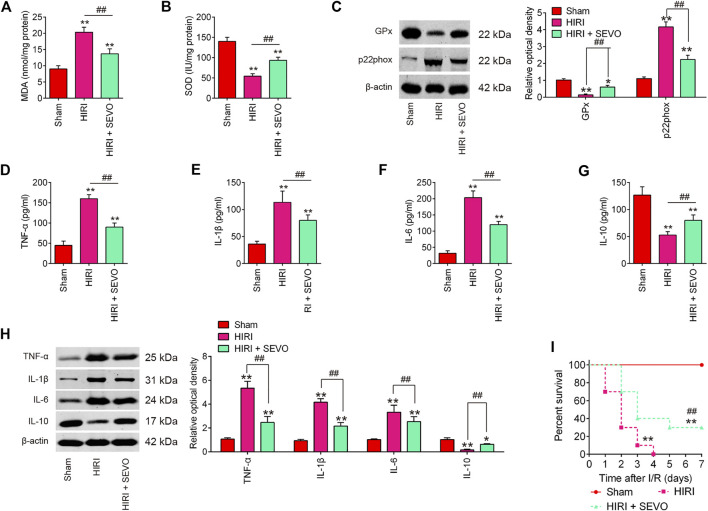
Sevoflurane postconditioning attenuates oxidant stress and inflammatory response. Mice were subjected to ischemia, then post-treatment with inhalation of 2% Sevo for 2 h immediately at the onset of reperfusion. **(A, B)** The contents of MDA and SOD were measured by ELISA assays (*n* = 6/group). **(C)** The expressions of GPx and p22phox were detected by Western Blot (*n* = 6/group). **(D–G)** The inflammatory cytokines including TNF-α, IL-1β, IL-6, and IL-10, were evaluated by ELISA assays (*n* = 6/group). **(H)** The protein expressions of TNF-α, IL-1β, IL-6, and IL-10 were determined by Western Blot (*n* = 6/group). **(I)** Animal survival was recorded at the indicated time points (*n* = 10/group). All data were measurement data which were expressed as mean ± standard deviation and analyzed by one-way analysis of variance. *p < 0.05, **p < 0.01 vs. Sham group; ^##^ *p* < 0.01 vs. HIRI group.

To determine whether Sevo postconditioning exerts the protective effects against hepatic I/R injury through regulation of oxidative stress, the oxidative stress biomarkers MDA and the antioxidant marker SOD were evaluated. High levels of MDA, and low levels of SOD were observed in HIRI group compared with Sham group, while level of MDA was significantly decreased, and level of SOD was markedly increased after Sevo postconditioning compared with HIRI group (Figures 2A,B). Meanwhile, the protein levels of the antioxidative enzyme, glutathione peroxidase (GPx) and a major enzyme in reactive oxygen species (ROS) generation, p22phox were detected by Western Blot. The results showed that the expression levels of GPx protein were significantly decreased, while the expression levels of p22phox protein were markedly increased in HIRI group compared with Sham group. However, Sevo postconditioning significantly reversed the I/R-induced changes (Figure 2C). All these data suggest that Sevo postconditioning could improve the oxidative stress caused by I/R *in vivo.*

It is well-known that excessive inflammatory response also has important roles in the development of hepatic I/R injury (Yu et al., 2016). Therefore, the inflammatory cytokines, such as TNF-α, IL-1β, IL-6, and IL-10 were assayed to evaluate if Sevo postconditioning also attenuates I/R induced inflammatory response. As shown in Figures 2D–G, hepatic I/R markedly increased the serum levels of pro-inflammatory cytokines TNF-α, IL-1β, and IL-6, and decreased the serum levels of anti-inflammatory cytokine, IL-10, compared with Sham group. However, Sevo postconditioning significantly reduced the production of these pro-inflammatory cytokines, and induced the serum levels of IL-10 compared with HIRI group. Similar results were observed in the proteins levels of TNF-α, IL-1β, IL-6, and IL-10, as determined by Western Blot (Figure 2H). In addition, all of the mice in HIRI group succumbed within 4 days whilst the survival rate of mice in the HIRI + Sevo group was significantly higher than that of the HIRI group (Figure 2I). All these data suggest that Sevo postconditioning improves hepatic I/R injury through regulation of oxidative stress and inflammatory response.

### Sevo Postconditioning Reverts I/R-Induced Downregulation of miR-142

**FIGURE 3:**
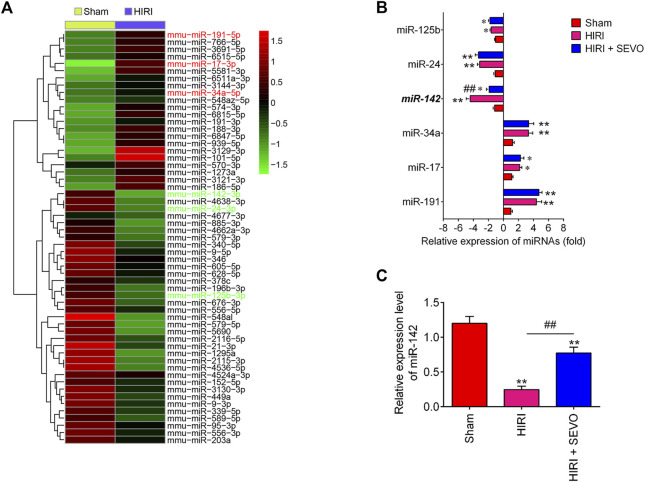
Sevoflurane postconditioning reverts I/R-induced downregulation of miR-142. **(A)** Heat map analysis of the miRNAs expression of groups (HIRI and Sham) was created using a method of hierarchical clustering by GeneSpring GX, version 7.3. Rows: Groups; Columns: microRNAs; Color key indicates microRNA expression value, red: Highest, green: Lowest. Microarray data obtained from GEO, GSE number is GSE72314. **(B)** The expression levels of miR-125b, miR-24, miR-142, miR-34a, miR-17, and miR-191 were detected in Sham, HIRI, and HIRI + Sevo groups by qRT-PCR (*n* = 6/group). **(C)** The expression of miR-142 was measured in Sham, HIRI, and HIRI + Sevo groups by qRT-PCR in 6 mice/group. All data were measurement data which were expressed as mean ± standard deviation and analyzed by one-way analysis of variance. *p < 0.05, **p < 0.01 vs. Sham group; ^##^ *p* < 0.01 vs. HIRI group.

Providing that miRNAs have key roles in oxidative stress, apoptosis and inflammatory response (Dando et al., 2015; Shen et al., 2020), we investigated whether the anti-oxidative, anti-apoptotic and anti-inflammatory activities of Sevo postconditioning occurs by altering the expression of miRNAs. The gene expression datasets of hepatic I/R injury were retrieved from GSE72314. 57 differentially expressed-miRNAs were observed between HIRI and Sham group (Figure 3A). In our microarray data, miR-125b, miR-24, and miR-142 were significantly decreased, while miR-34a, miR-17, and miR-191 were increased in HIRI group compared with Sham group, which are consistent with previous studies (Huang et al., 2019; Li et al., 2016; Pan et al., 2019; Qin et al., 2018; Shen et al., 2020), suggesting the reliability of our microarray results. Notably, the decreased expression of miR-142 caused by hepatic I/R injury were significantly reversed by Sevo postconditioning; however, no significant changes were observed for miR-125b, miR-24, miR-34a, miR-17 and miR-191 expressions (Figure 3B). Previous studies have demonstrated that miR-142 plays an important role in various tissue I/R injury including liver. For example, Li et al. reported that miR-142 may be a potential agent for protection against cerebral I/R injury through down-regulation of FBXO3 (Li and Ma, 2020). Wang et al. showed that miR-142-3p attenuated myocardial I/R injury by inhibiting the apoptosis of cardiomyocytes and cardiac fibrosis (Wang et al., 2016). Notably, a recent study demonstrated that miR-142-5p could relieve hepatic I/R injury through alleviating apoptosis and inflammation in mice (Li et al., 2020). Therefore, we chose miR-142 for further investigation. Subsequently, we detected the expression levels of miR-142 in liver tissues of mice after Sevo postconditioning. As shown in Figure 3C, the miR-142 expression levels were significantly reduced in HIRI group, compared with Sham group, however, this inhibitory effect was dramatically reversed by Sevo postconditioning. Previous studies have reported that miR-142 plays important roles in regulating I/R injury in other organs, such as kidney and heart (Zhu et al., 2018; Zhao Z. et al., 2019); thus, we proposed that Sevo postconditioning may improve hepatic I/R injury by reverting I/R-induced downregulation of miR-142.

### Sevo Postconditioning Improves Hepatic I/R Injury by Regulating miR-142 Expression

**FIGURE 4:**
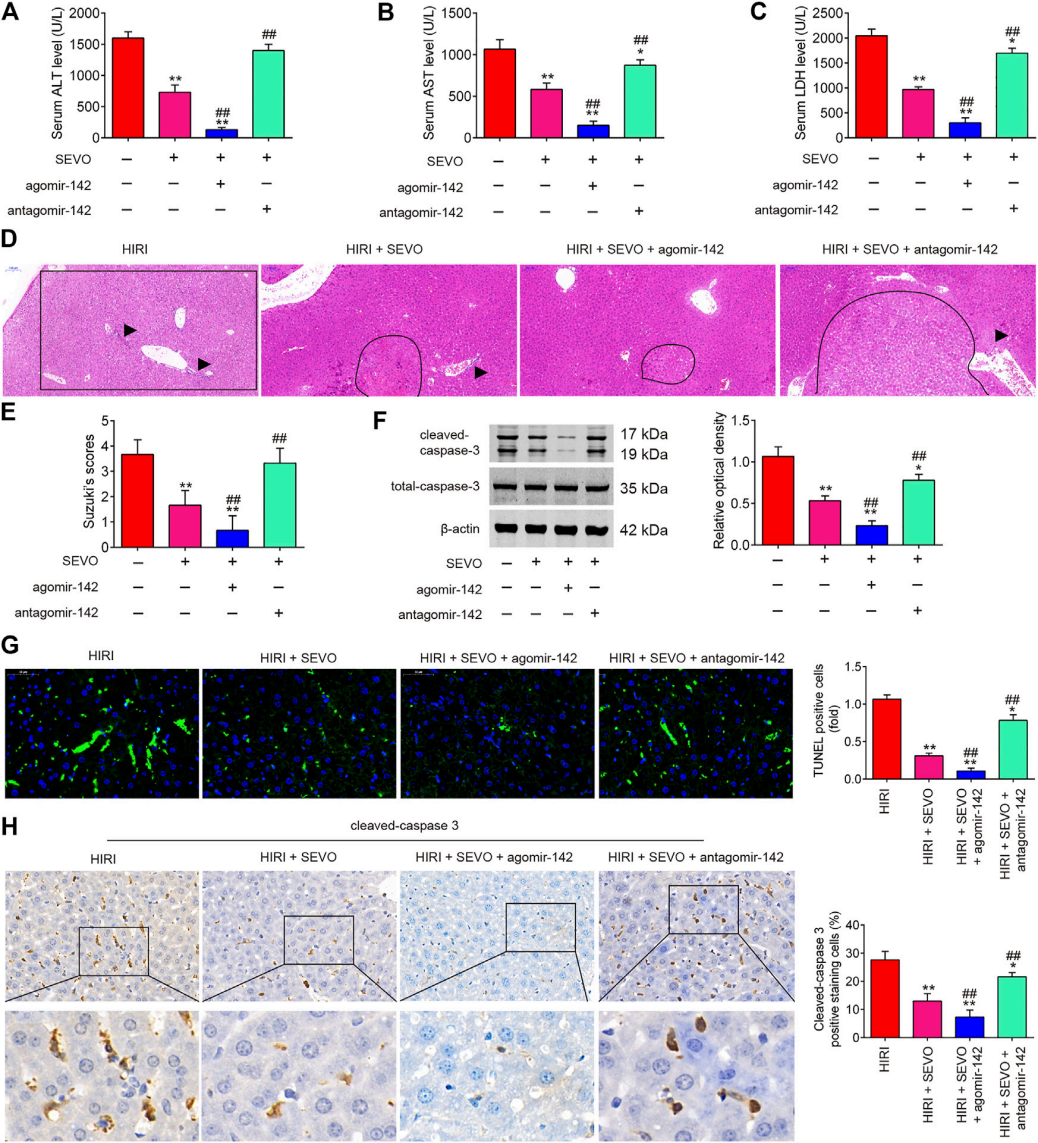
Sevoflurane postconditioning decreases hepatic I/R injury through regulation of miR-142. The agomiR-142 and antagomiR-142 were injected into mice (*n* = 6/group), and then the mice were subjected to ischemia, post-treatment with inhalation of 2% Sevo for 2 h immediately at the onset of reperfusion. **(A–C)** Liver enzyme assays were performed in the four groups: HIRI, HIRI + Sevo, HIRI + Sevo + agomiR-142, and HIRI + Sevo + antagomiR-142 groups (*n* = 6/group) (A: Serum ALT levels; B: Serum AST levels; C: Serum LDH levels). **(D, E)** Hepatic tissues of mice in each group examined by HE staining and was graded using the Suzuki score. The inflammatory cell infiltration is indicated with black arrow and necrotic areas is indicated with curve. **(F)** The expression of cleaved caspase-3 was detected by Western Blot. **(G)** The apoptosis was determined by TUNEL staining assay. **(H)** The expression of caspase-3 was measured by IHC. All data were measurement data which were expressed as mean ± standard deviation and analyzed by one-way analysis of variance. *p < 0.05, **p < 0.01 vs. HIRI group; ^##^ *p* < 0.01 vs. HIRI + Sevo group.

It was further evaluated if miR-142 participates in the protective effect of Sevo postconditioning on hepatic I/R injury. AgomiR-142 and antagomiR-142 were injected into mice, and then the mice were subjected to ischemia, post-treatment with inhalation of 2% Sevo for 2 h immediately at the onset of reperfusion. As shown in Figures 4A–E, the liver functions were obviously improved after Sevo postconditioning, as determined by the histopathological studies and alterations in liver enzymes such as AST, ALT and LDH; however, this improvement was enhanced by agomiR-142, while reversed by antagomiR-142. Western blot analysis revealed that the expression levels of cleaved caspase-3 were significantly reduced by Sevo postconditioning compared with HIRI group, but it was even lower in HIRI + Sevo + agomiR-142 group, while the decreased expression of cleaved caspase 3 in HIRI + Sevo group was significantly increased in HIRI + Sevo + antagomiR-142 group (Figure 4F). Similar effects were observed in TUNEL-positive hepatocytes and the expression levels of cleaved caspase 3, as determined by TUNEL staining and IHC, respectively (Figures 4G,H). Taken together, Sevo postconditioning may ameliorate hepatic I/R-induced injury in mice through upregulating miR-142 expression.

### Sevo Postconditioning Exhibits Anti-oxidative and Anti-inflammatory Activities by Regulating miR-142

**FIGURE 5:**
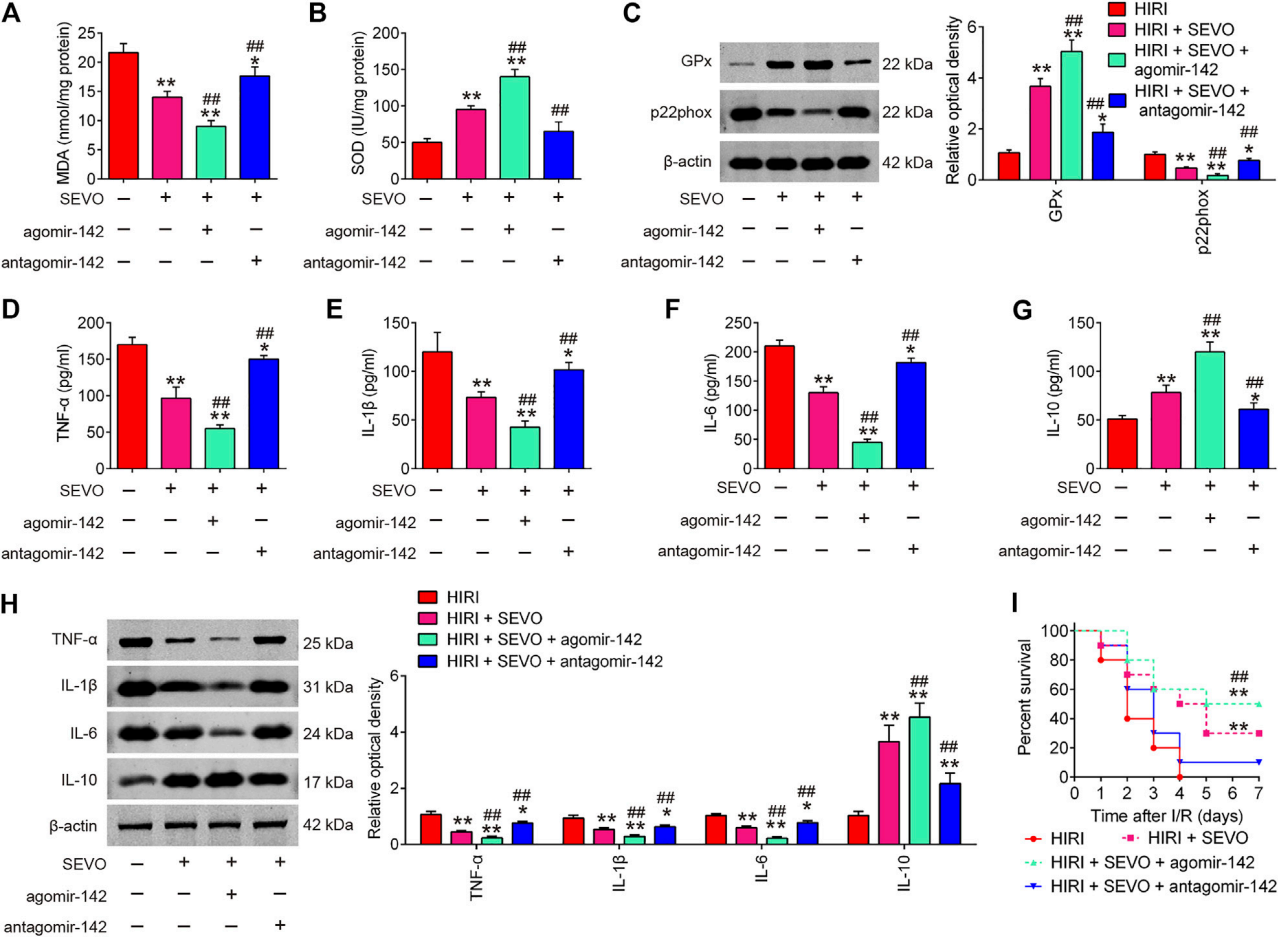
Sevoflurane postconditioning attenuates oxidant stress and inflammatory response through regulation of miR-142. The agomiR-142 and antagomiR-142 were injected into mice, and then the mice were subjected to ischemia, post-treatment with inhalation of 2% Sevo for 2 h immediately at the onset of reperfusion. **(A, B)** The contents of MDA and SOD were measured by ELISA assays (*n* = 6/group). **(C)** The expressions of GPx and p22phox were detected by Western Blot (*n* = 6/group). **(D–G)** The inflammatory cytokines including TNF-α, IL-1β, IL-6 and IL-10, were evaluated by ELISA assays (*n* = 6/group). **(H)** The protein expressions of TNF-α, IL-1β, IL-6, and IL-10 were determined by Western Blot (*n* = 6/group). **(I)** Animal survival was recorded at the indicated time points (*n* = 10/group). All data were measurement data which were expressed as mean ± standard deviation and analyzed by one-way analysis of variance. *p < 0.05, **p < 0.01 vs. HIRI group; ^##^ *p* < 0.01 vs. HIRI + Sevo group.

Given the key roles of miR-142 in the oxidative stress and inflammatory response in various types of diseases (Li and Ma, 2019; Su et al., 2019), we determine to investigate whether Sevo postconditioning exerts its anti-oxidative and anti-inflammatory activities through regulating miR-142. As illustrated in Figures 5A,B, low serum levels of MDA concentration and high serum levels of SOD were observed in the Sevo + HIRI group compared with HIRI group. Furthermore, we found that agomiR-142 enhanced the anti-oxidative activity of Sevo, while antagomiR-142 significantly attenuated the anti-oxidative activity in HIRI mice. Similar effects were observed in GPx and p22phox protein expressions (Figure 5C).

In addition, the inflammatory response was evaluated by ELISA assay. As shown in Figures 5D–G, the productions of TNF-α, IL-1β, and IL-6 were significantly decreased; IL-10 was markedly increased in Sevo + HIRI group compared with HIRI group. As expected, the anti-inflammatory effects of Sevo was enhanced by agomiR-142, whereas the anti-inflammatory effects of Sevo were significantly attenuated by antagomiR-142 in HIRI mice. Similar effects were observed in TNF-α, IL-1β, IL-6, and IL-10 protein expressions (Figure 5H). In addition, we found that agomiR-142 significantly enhanced the improvement of Sevo in the survival rate of mice, while the survival rate of mice in HIRI + Sevo + antagomir-142 group was significantly lower than that of the HIRI + Sevo group (Figure 5I). All these data indicate that Sevo postconditioning suppresses oxidative stress and inflammatory response *via* miR-142 upregulation.

### HMGB1 Is a Direct Target of miR-142 *in vitro* and *in vivo*

**FIGURE 6:**
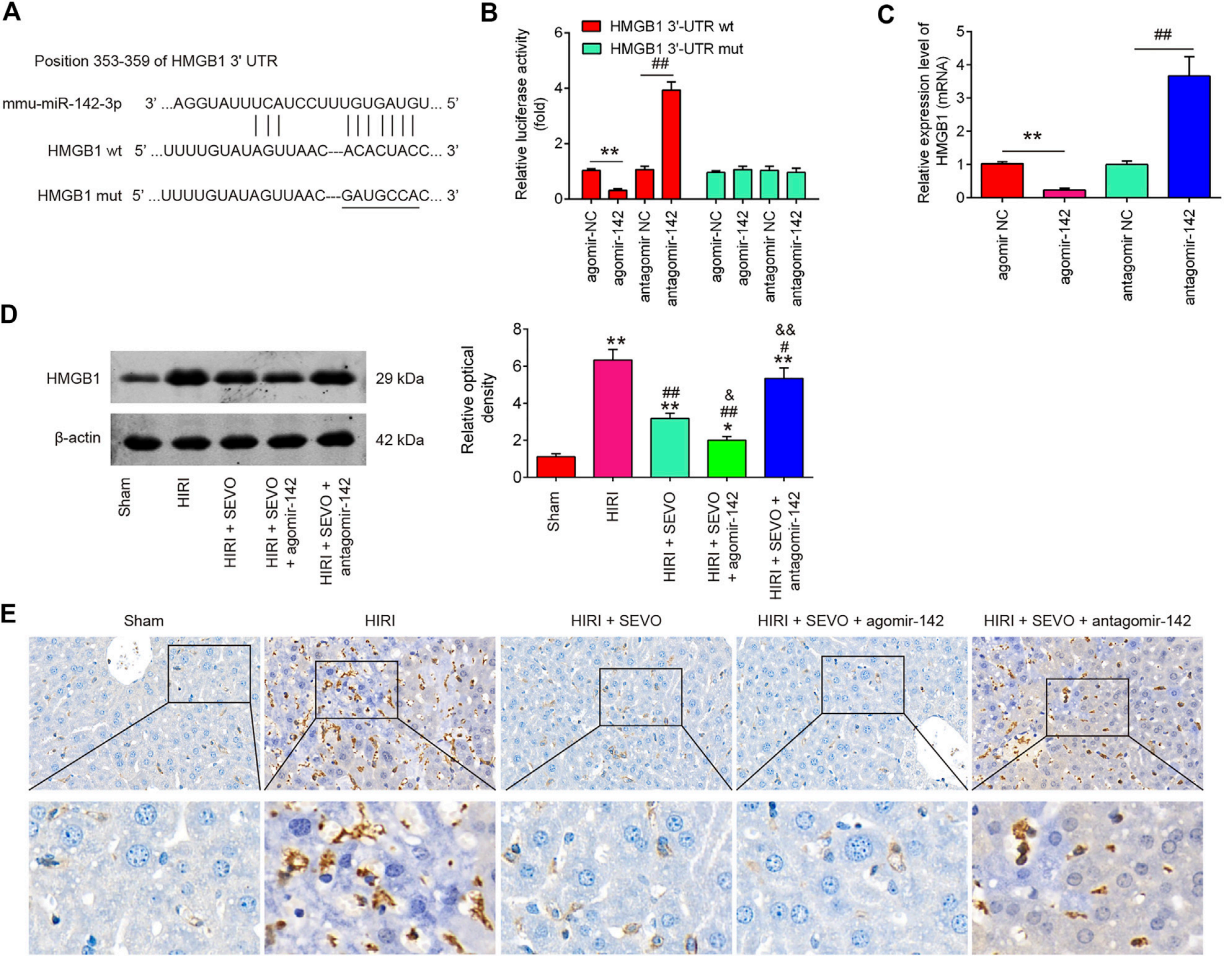
HMGB1 is a direct target of miR-142. **(A)** miR-142-binding sequences in the 3′-UTR of HMGB1 and mutated sites in 3′-UTR of HMGB1. **(B)** AgomiR-142 suppressed the luciferase activities of constructs containing the 3′-UTR segment of HMGB1, while antagomiR-142 significantly increased the luciferase activities of constructs containing the 3′-UTR segment of HMGB1 (*n* = 3). **(C)** The mRNA expressions of HMGB1 were detected by qRT-PCR after treatment with agomiR-142 or antagomiR-142 in NCTC1469 cells (*n* = 3). All data were measurement data which were expressed as mean ± standard deviation and analyzed by one-way analysis of variance. **p < 0.01 vs. agomiR-NC group; ^##^ *p* < 0.01 vs. antagomiR-NC group. The agomiR-142 and antagomiR-142 were injected into mice (*n* = 6/group), and then the mice were subjected to ischemia, post-treatment with inhalation of 2% Sevo for 2 h immediately at the onset of reperfusion. **(D, E)** The protein expression of HMGB1 was measured by Western Blot and IHC. All data were measurement data which were expressed as mean ± standard deviation and analyzed by one-way analysis of variance. *p < 0.05, **p < 0.01 vs. Sham group; ^##^ *p* < 0.01 vs. HIRI group. and *p* < 0.01 vs. HIRI + Sevo group.

To identify the target gene of miR-142, bioinformatic tools (Targetscan and mirbase) were performed. As shown in Figure 6A, miR-142 was predicted as a putative miRNA targeting HMGB1. Meanwhile, a luciferase reporter assay was conducted to confirm the interaction of miR-142 and HMGB1. It was shown that agomiR-142 significantly reduced, whereas antagomiR-142 markedly increased the relative luciferase activity of HMGB1 3′-UTR wt. However, no significant differences were found in the luciferase activity when NCTC 1469 cells were co-transfected with HGMB1-3′UTR mut reporter and agomiR-142/antagomiR-142 (Figure 6B). Furthermore, the mRNA expression of HGMB1 was significantly down-regulated in the agomiR-142 group compared to agomiR-NC group and increased in antagomiR-142 compared with antagomir-NC group in NCTC 1469 cells (Figure 6C). We also found that Hepatic I/R injury caused dramatic induction of HGMB1 levels, compared with Sham group, however, the HGMB1 levels were significantly reduced after Sevo postconditioning in mice undergoing hepatic I/R. Furthermore, the much lower protein levels of HGMB1 was observed in HIRI + Sevo + agomiR-142 promoted the inhibitory effect of Sevo postconditioning on in mice undergoing hepatic I/R, whereas antagomiR-142 reversed those effects (Figure 6D). Similar results in the protein expression levels of HGMB1 were observed using IHC assay (Figure 6E). All these results suggest that HGMB1 may be a functional target of miR-142 *in vitro* and *in vivo.*

### Sevo Postconditioning Blocked TLR4/MyD88/NF-κB Signaling Pathway Activation

**FIGURE 7:**
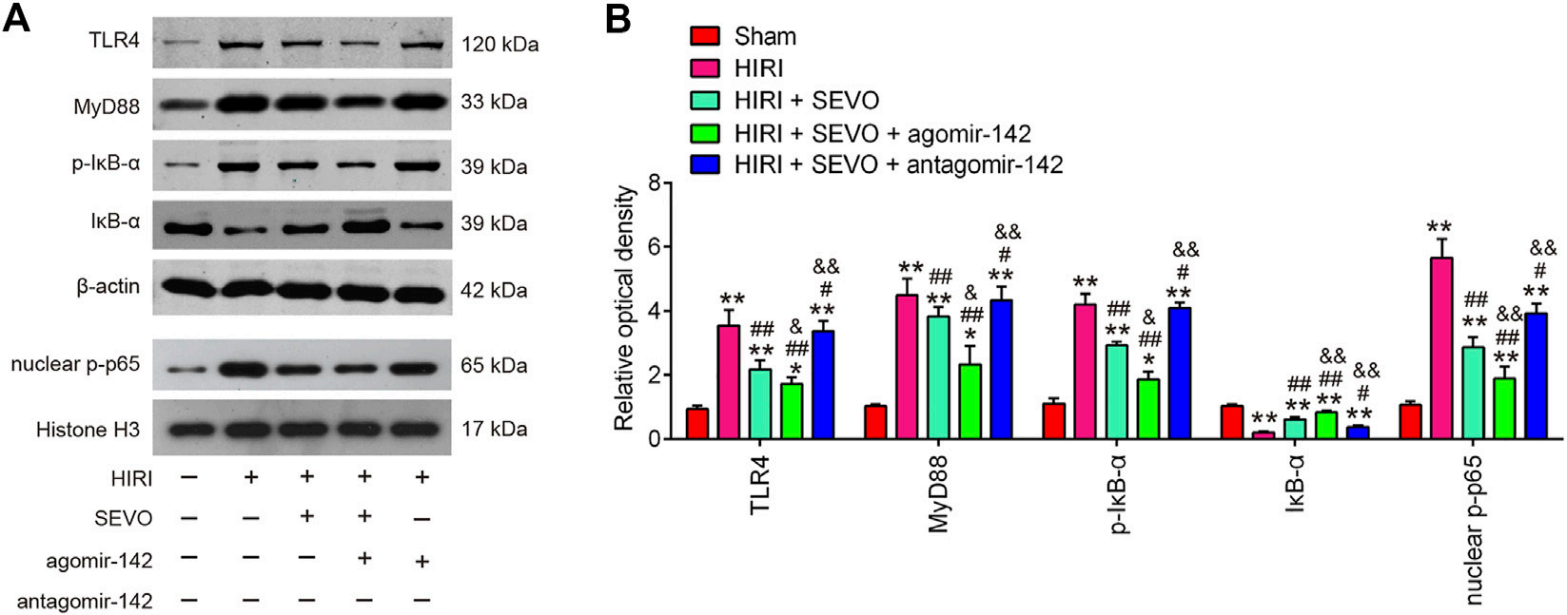
Sevo postconditioning blocked the activation of TLR4/MyD88/NF-κB signaling pathway. The agomiR-142 and antagomiR-142 were injected into mice (*n* = 6/group), and then the mice were subjected to ischemia, post-treatment with inhalation of 2% Sevo for 2 h immediately at the onset of reperfusion. **(A, B)** The protein expressions of TLR4, MyD88, p-IκB-α and nuclear-p-p65 were detected by Western Blot. All data were measurement data which were expressed as mean ± standard deviation and analyzed by one-way analysis of variance. *p < 0.05, **p < 0.01 vs. Sham group; ^#^ *p* < 0.05, ^##^ *p* < 0.01 vs. HIRI group. and *p* < 0.01 vs. HIRI + Sevo group.

Toll-like receptor 4 (TLR4) is known to be responsible for HMGB1-induced inflammatory response (Yang et al., 2015). Thus, the interaction between the hepatic protective effect of Sevo postconditioning and TLR4 signaling pathway needs to be elucidated. Western blot analysis was performed to detect the protein levels of TLR4, MyD88, p-IκB-α and nuclear-p-p65 in the following groups: Sham, HIRI, HIRI + Sevo, HIRI + Sevo + agomiR-142, and HIRI + Sevo + antagomir-142. As shown in Figures 7A,B, hepatic I/R injury resulted in a significant induction of TLR4, MyD88, p-IκB-α and nuclear-p-p65 expressions, compared with Sham group. However, all these proteins were significantly reduced in hepatic I/R mice after Sevo postconditioning. Moreover, agomiR-142 enhanced the inhibitory effect of Sevo postconditioning on all these proteins levels in hepatic I/R mice, whereas antagomiR-142 reversed this effect. All data suggest that Sevo postconditioning may suppress inflammatory response through inhibiting the TLR4/MyD88/NF-κB signaling pathway.

### Sevo Postconditioning Exerts its Protective Effect Against Hepatic I/R Injury Through Inhibiting the TLR4/MyD88/NF-κB Signaling Pathway *in vitro*

**FIGURE 8:**
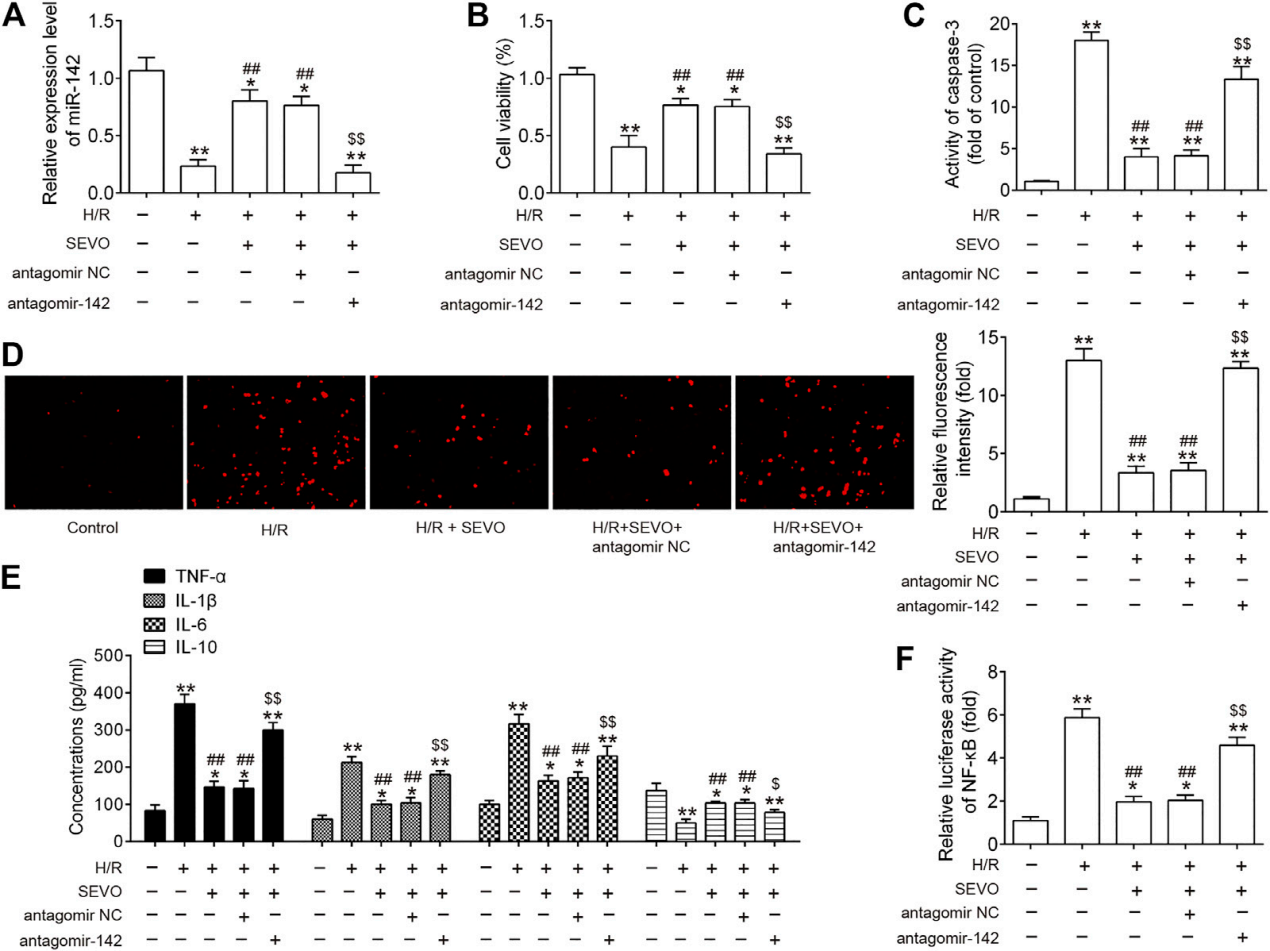
Sevo postconditioning exerts its protective effect against hepatic I/R injury through inhibiting the TLR4/MyD88/NF-κB signaling pathway *in vitro.* Cells were transfected with antagomir-NC/antagomir-142 (100 nM) for 24 h, and then subjected to H/R for 6 h. Before hypoxia treatment, cells were placed in a semi-airtight container and 2% sevoflurane was used for 2 h of pre-treatment. **(A)** The expression levels of miR-142 was measured by qRT-PCR. **(B)** Cell viability was determined by CCK-8 assay. **(C)** The activity of caspase-3 was measured by a commercial kit. **(D)** ROS production was assessed by DCFH-DA assay. **(E)** The inflammatory and anti-inflammatory cytokines including TNF-α, IL-1β, IL-6, and IL-10, were evaluated by ELISA assays. **(F)** Luciferase activity of NF-κB was quantified using the Promega luciferase assay kit on a luminometer. All data were measurement data which were expressed as mean ± standard deviation and analyzed by one-way analysis of variance. *p < 0.05, **p < 0.01 vs. Control group; ^#^ *p* < 0.05, ^##^ *p* < 0.01 vs. H/R group; ^$^ *p* < 0.05, ^$$^ *p* < 0.01 vs. H/R + Sevo + antagomir NC group.

To further understand the protective mechanisms of Sevo postconditioning against hepatic I/R injury, mouse embryonic hepatocytes BNL CL.2 cells exposed to hypoxia and reoxygenation (H/R) were used to mimic hepatic I/R injury *in vitro*. The present study knocked down the expression of miR-142 *via* transfection of antagomir-142 prior to H/R induction and Sevo treatment, and then observed the alterations in the effects of Sevo in this cell model. Firstly, it was observed that the expression of miR-142 was significantly decreased following antagomir-142 transfection in BNL CL.2 cells under I/R injury (Figure 8A). CCK-8 assay showed that the cell viability was significantly decreased in H/R group compared with control group, while the cell viability was significantly increased in the Sevo + H/R group compared with H/R group. However, the promoting effect of Sevo on cell viability was attenuated by antagomir-142 transfection (Figure 8B). In the next step, the caspase 3 activity was determined of BNL CL.2 cells in each group. As shown in Figure 8C, the caspase 3 activity of Sevo + H/R group was significantly lower than that of H/R group, while the caspase 3 activity of BNL CL.2 cells was significantly increased in the Sevo + H/R group + antagomiR-142 group compared with Sevo + H/R group. According to the DCFDA assay, H/R caused an increase in ROS production, while Sevo treatment significantly reduced ROS production. Similarly, antagomiR-142 attenuated the inhibitory effects of Sevo on the ROS production (Figure 8D). Moreover, the levels of pro-inflammatory cytokines TNF-α, IL-1β, IL-6, and anti-inflammatory cytokine, IL-10 were detected by ELISA assays. In line with the results *in vivo*, Sevo treatment suppressed the inflammatory response caused by I/R, whereas antagomiR-142 abolished its anti-inflammatory activity of Sevo (Figure 8E). We also measured the NF-κB activity under this condition. It was shown that NF-κB activity was significantly increased in H/R group compared with control group, and this promotional effect was inhibited by Sevo. However, inhibition of miR-142 by the antagomir-142 reversed the inhibitory effects of Sevo on the NF-κB activity (Figure 8F). These results suggest that Sevo-postconditioning improves hepatic I/R injury by regulating miR-142/NF-κB axis *in vitro*.

## Discussion

**FIGURE 9:**
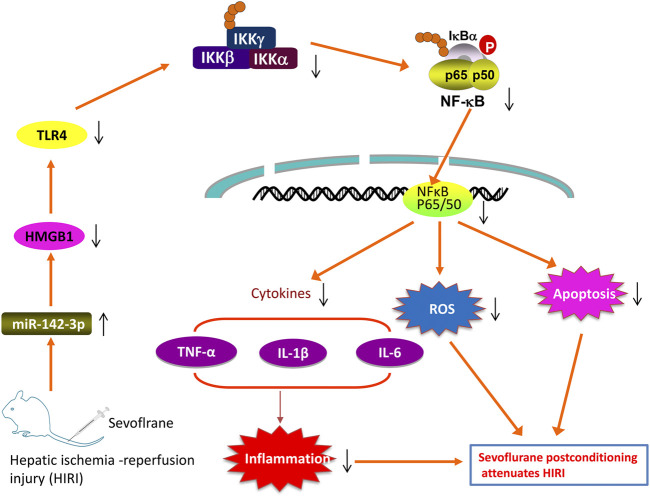
Schematic diagrams showing that Sevo postconditioning counteracts the downregulation of miR-142 provoked by I/R, in turn decreased the expression of HMGB1, blocking TLR4/NF-κB pathway activation, thus improving hepatic I/R injury, as evidenced by the suppression of inflammatory responses, oxidant stress and cell apoptosis.

In the present study, we found that Sevo postconditioning improves hepatic I/R injury through the modulation of oxidative stress, inflammatory response and apoptosis. Additional experiments suggest that Sevo postconditioning counteracts the downregulation of miR-142 provoked by I/R, which in turn reduces HGMB1 levels to subsequently block the activation of TLR4/MyD88/NF-κB signaling pathway (Figure 9).

Sevo is one of the commonly used inhalation anesthetic, similar as isoflurane and desflurane (Wu et al., 2017). Both Sevo preconditioning and postconditioning have been proven to attenuate injury in various experimental animal models subjected to ischemia-reperfusion (I/R) (Zhu et al., 2017; Liu T.-J. et al., 2018). Sevo induced these protective properties are closely associated with its anti-inflammatory and anti-apoptotic activities (Ohsumi et al., 2017). To date, Sevo has been widely used during hepatobiliary surgery and was reported to exhibit preconditioning properties against hepatic IR injury (Beck-Schimmer et al., 2008; Xu et al., 2019). For example, a recent study has demonstrated that Sevo preconditioning exerts protective effects on liver I/R injury through suppressing inflammation in rats (Liao et al., 2019). However, its postconditioning properties remain unknown. In this study, we found that Sevo postconditioning could improve the hepatic I/R injury through inhibiting oxidative stress, apoptosis and inflammatory response in mice. However, the possible molecular mechanism requires further investigation to be fully understood.

Accumulating studies have revealed important role of miRNAs and their target genes in regulating hepatic I/R injury in animal or cell models (Tang et al., 2019; Shen et al., 2020). For example, Zheng et al. have found that miR-148a may mitigate hepatic I/R injury by ameliorating TLR4-mediated inflammatory response *via* targeting CaMKIIα *in vitro* and *in vivo* (Zheng et al., 2018). Xiong et al. have shown that miR-93 upregulation improved liver function through depressing improving apoptosis and suppressing inflammatory response (Xiong et al., 2018). Additionally, the protective effects of Sevo against I/R injury were well regulated by miRNAs (Wu et al., 2016; Wenlan et al., 2018; Zhao Y.-B. et al., 2019). For example, Zhang et al. reported that Sevo preconditioning exhibited an effective neuroprotective against cerebral I/R possibly by inhibiting miR-181a in middle cerebral artery occlusion (MCAO) rat model (Zhang et al., 2019b). In this study, microarray screening revealed miR-142 was one of the major miRNAs that were upregulated in mice by Sevo treatment. Previous studies have demonstrated that miR-142 improved I/R injury in different organs including heart (Su et al., 2019) and kidney (Zhu et al., 2018); however, whether miR-142 is involved in the protective mechanisms of Sevo postconditioning in hepatic I/R injury remains unknown. In our findings, it was confirmed that the agomir-142 injection enhanced the protective effects when compared with Sevo, while the antagomir-142 injection suppressed the therapeutic effects of Sevo in mice. In addition, we also demonstrate that Sevo postconditioning exerts its protective effect against hepatic I/R injury through increasing miR-142 expression in a cellular model of simulated I/R in NCTC 1469 cell line. Taken together, these results indicate that miR-142 upregulation may contribute to Sevo postconditioning-induced protective effects against hepatic I/R injury.

The elucidation of functional targets of miRNAs is one of the best ways to understand miRNA function. HMGB1 is a well-known pro-inflammatory mediator, which is involved in various pathological conditions (Yao et al., 2016; Yang et al., 2018). One recent study has shown that blockade of extracellular HMGB1 protects mice from acute liver injury (Gong et al., 2010). Similarly, another study from Chen et al. have demonstrated that HMGB1 knockdown significantly attenuated carbon tetrachloride (CCl4)-induced hepatic injury through regulation of oxidative stress and inflammatory response (Chen et al., 2014). In our study, HGMB1 was confirmed as a direct target of miR-142 using bioinformatic tools and luciferase reporter assay, respectively, which is consistent with the Su' findings (Su et al., 2019). We found that Sevo partially counteracts the downregulation of miR-142 provoked by I/R, thus reduces the expression of HGMB1 in mice.

As we known, HMGB1 binds to multiple receptors such as the transmembrane toll-like receptors (TLRs) and thus activates NF-κB signaling pathway through a MyD88-dependent mechanism (El Gazzar, 2007; He et al., 2012). The NF-κB in turn transcriptionally promotes the productions of inflammatory cytokines, such as TNF-α, IL-1β, and IL-6. It has previously been shown that the TLR4-MyD88-NF-κB pathway is a key signaling pathway in hepatic I/R injury (Xu et al., 2016). Therefore, blocking of this pathway may be used as an effect therapeutic method to protect against hepatic I/R injury (Du et al., 2019). Based on above studies, we propose that Sevo postconditioning alleviated hepatic I/R injury by inhibiting the activation of HMGB1/TLR4-MyD88-NF-κB signaling pathway by upregulating miR-142 expression. As expected, our results showed that Sevo postconditioning significantly blocked the activated TLR4-MyD88-NF-κB signaling pathway caused by I/R injury. Furthermore, miR-142 upregulation enhanced the inhibitory effects of Sevo postconditioning on this pathway, whereas miR-142 inhibition reversed these effects. Taken together, these data demonstrated that Sevo postconditioning improves hepatic I/R injury *via* the miR-142/HMGB1/TLR4/NF-κB axis.

In conclusion, our results demonstrate that Sevo postconditioning exhibits an effective protection against hepatic I/R injury, which was largely dependent on the upregulation of miR-142 *via* suppressing the I/R-activated TLR4/MyD88/NF-κB signaling pathway. Our findings suggest that Sevo be a valuable alternative anaesthetic agent in liver transplantation and major liver surgeries.

## Data Availability

The original contributions presented in the study are included in the article/Supplementary Material, further inquiries can be directed to the corresponding authors.
